# Cognition in type 2 diabetes: Association with vascular risk factors, complications of diabetes and depression

**DOI:** 10.4103/0972-2327.48848

**Published:** 2009

**Authors:** Thomas Iype, Sivaraman K. Shaji, Ajitha Balakrishnan, Deepak Charles, Anu A. Varghese, T. P. Antony

**Affiliations:** Department of Neurology, Medical College, Thiruvananthapuram, India; 1 Department of Psychiatry, Medical College, Thrissur, India; 2 Department of Obstetrics and Gynaecology, Medical College, Kozhikode, India; 3 Department of Medicine, Amala Institute of Medical Sciences. Thrissur, India

**Keywords:** Cognition, depression, diabetes mellitus, hypertension, vascular risk factor

## Abstract

**Background:**

The role of variables like duration of diabetes, diabetic control and microvascular complications in the causation of cognitive decline in patients with type 2 diabetes is not well studied. The contribution of hypertension to the cognitive decline in nondemented diabetic patients is unclear.

**Aims:**

We wanted to see if cognition in patients with type 2 diabetes is associated with the duration of diabetes, control of diabetes, complications of diabetes, vascular risk factors, or depression. We also looked at association of noncompliance with cognition, and depression.

**Settings and Design:**

We recruited ambulant patients with type 2 diabetes who are 55 years or more in age from the weekly diabetic clinic. We excluded patients with past history of stroke.

**Methods and Material:**

We selected the time taken for the Trial A test, delayed recall on ten-word list from Consortium to Establish a Registry for Alzheimer's Disease (CERAD), Rowland Universal Dementia Assessment Scale (RUDAS) and Centre for Epidemiologic Studies Depression scale (CES-D) screening instrument to assess these patients.

**Statistical Analysis Used:**

We utilized mean, standard deviation, Chi-square test and Pearson's correlation for statistical analysis. We considered *P* < 0.05 to be significant.

**Results:**

RUDAS scores inversely correlated (*r* = −0.360) with CES-D scores (*P* = 0.002). Scores of the screening instrument for depression, the CES-D was associated with the duration of diabetes mellitus (*P* = 0.018), fasting blood glucose (*P* = 0.029) as well as with 2-hour post prandial blood glucose (*P* = 0.017).

**Conclusions:**

There is correlation between depression and global cognitive score. Depression seems to be associated with duration of diabetes and control of diabetes.

## Introduction

A systematic review emphasized the contradictory data on cognition and diabetes.[1] The cognitive impairment when present in type 2 diabetes mainly produces mental slowing, reduced mental flexibility and impaired learning and memory. The evidence associating hypertension with cognitive decline in diabetes patients without dementia is conflicting.[2,3] The role of diabetes-related variables such as the duration of diabetes, diabetic control and microvascular complications in causing cognitive decline in diabetics is not well studied.[1]

We wanted to find out the factors associated with cognitive decline in type 2 diabetic patients. We also looked for the association of independent variables such as the duration of diabetes, control of diabetes, complications of diabetes, other vascular risk factors and comorbid conditions such as depression with cognitive decline in diabetics. Association of noncompliance with cognitive decline and depression was also studied. If such an association is present, it would have important implications in management. Appropriate intervention to control depression, hypertension and vascular risk factors, would then be useful in preventing or reversing cognitive decline in patients with type 2 diabetes. If noncompliance is associated with depression, then treating depression can potentially reduce noncompliance.

## Materials and Methods

After obtaining informed consent, we recruited consecutive ambulant patients with type 2 diabetes above the age of 55 years attending the weekly diabetic clinic at Medical College. We excluded patients with a past history of stroke. We recorded the demographic data, educational background, complications of diabetes, other vascular risk factors and compliance to treatment. Blood pressure was measured using mercury manometer in the lying position on the day of assessment after 10 minutes of rest. Body mass index was calculated as the ratio of weight in kilograms to square of height in meters. Hypertension was diagnosed during the previous follow up in the diabetic clinic and where on antihypertensives. We diagnosed peripheral neuropathy based on history of paraesthesia, absent ankle jerk or impaired sensations such as pain, temperature, touch or vibration sense. Absence of peripheral pulses in the lower limb other than dorsalis pedis along with limb claudication suggested peripheral vascular disease. A drop of 20 mm Hg systolic blood pressure or a fall of 10 mm Hg of diastolic blood pressure immediately or after 3 minutes of standing indicated autonomic nervous system affection. One of the authors (TI) examined the fundi to diagnose diabetic retinopathy. Urine albumin creatinine ratio > 0.3 suggested diabetic nephropathy. History of smoking was recorded. We made a diagnosis of coronary artery disease from history and electrocardiograph. We elicited history of noncompliance from the best guess of the approximate number of days of possible default in the last 1 year. We arbitrarily diagnosed noncompliance if patients defaulted more than 2.5% of the days in the previous one year. We used fasting blood glucose (<110 mg/dl) and post-prandial (<140 mg/dl) blood glucose to determine control of diabetes mellitus on the day of assessment. On the day of assessment, a systolic blood pressure of < 135 mm Hg and a diastolic blood pressure of < 85 mm Hg indicated control of blood pressure.

From the expected pattern of cognitive decline in diabetics, we selected the time taken for the Trial A test and the delayed recall on ten-word list from Consortium to Establish a Registry for Alzheimer's Disease (CERAD).[4] In addition, we assessed global cognition using education and culture fair Rowland Universal Dementia Assessment Scale (RUDAS). A doctor blind to the demographic data, control of diabetes, complications of diabetes and the hypothesis of the study administered the tests. A nonpsychiatrist administered the Centre for Epidemiologic Studies Depression scale (CES-D)[5] screening instrument. We used mean, standard deviation, Chi-square test, Pearson's correlation and multivariate analysis for statistical analysis. *P* < 0.05 was considered to be significant. We categorized patients with diabetes into those with short and long duration of diabetes for analysis. Those patients who had duration of diabetes less than the mean duration of diabetes of the population were designated as having short-duration diabetes.

## Result

We examined 71 consecutive ambulant patients with diabetes above the age of 55 years on follow up between 17 December 2005 and 11 February 2006. They had a mean age of 64.87 years (SD: 6.87), mean formal education of 4.52 years (SD: 3.5) and male-female ratio of 1:1.22 (32:39). The mean duration of diabetes was 8.79 years (SD: 6.87).

The mean score for RUDAS was 23.7 (SD 3). Twenty patients (28%) had RUDAS score less than 23. RUDAS scores inversely correlated (*r* = −0.360) with CES-D scores (*P* = 0.002). RUDAS scores also correlated (*r* = 0.333) with delayed word list learning task (*P* = 0.005). RUDAS scores had inverse correlation (*r* = −0.262) with time taken for the trail-making A test (*P* = 0.041). RUDAS scores correlated (*r* = 0.25) with years of formal education (*P* = 0.035). Females were found to be more cognitively impaired (*P* = 0.002). We did not find any difference in the mean years of education between males and females. In multivariate analysis also, the result was same after controlling the effect of education. RUDAS scores were not associated with the duration of diabetes, hypertension, or diabetic complications such as peripheral neuropathy, retinopathy and postural hypotension. There was no association between RUDAS scores and blood glucose levels.

Both trail-making A test and delayed recall on 10 word list learning were not associated with duration of diabetes or hypertension. Three patients with blindness and seven illiterate patients could not complete the trail-making A test. Out of the 61 patients, 40 patients (65.6 %) took more than 120 s to complete it. None among the 71 patients had a delayed word list learning of less than 2.

None of the cognitive tests, RUDAS, trail-making A test and the word list learning were associated with percentage interruption of medication. There was no significant correlation between BMI and cognitive test scores.

Fifty (70.42%) out of the 71 patients had CES-D scores more than 3, the cut of score for depression. The CES-D score had an association with the duration of diabetes mellitus (*P* = 0.018), fasting blood glucose (*P* = 0.029) and 2-hour post prandial blood glucose (*P* = 0.017). However, there was no association with hypertension or complications of diabetes such as peripheral neuropathy, retinopathy and postural hypotension. Scores of CES-D was not correlated with that of TMT test and Delayed WLL

## Discussion

Even though it is a negative study, one of its strength is that this is one of the few studies which looked at diabetes-related variables such as the duration of diabetes, diabetic control, and micro-vascular complications of diabetes with the cognitive decline. We did not observe any correlation between cognitive decline and depression with noncompliance. Our study suggests that hypertension does not contribute to the cognitive decline.

The cognitive score as measured by RUDAS correlated with the CES-D depression score. Depression can cause reversible cognitive dysfunction especially in older people. This opens up the question whether treatment of depression is likely to improve cognition in the depression and diabetes. The correlation between depression duration of diabetes and blood sugar values is interesting. Does this mean that better control of diabetes will be associated with lesser prevalence of depression among patients with type 2 diabetes? Obviously, the large proportion of patients screened positive for depression indicates the need for intervention by a psychiatrist. Alternatively, the treating clinicians need to be trained in the diagnosis and management of depression in patients with type 2 diabetes.

This was a hospital based cross-sectional study, which can lead to selection bias and less generalizability. We know that the recorded duration of diabetes mellitus does not correlate with the onset of type 2 diabetes mellitus. We should have used glycosylated hemoglobin to correlate depression scores and cognitive scores rather than only the blood sugar values on the day of administration of the test. Similarly, 24-h blood pressure recording, left ventricular hypertrophy, assessed by echocardiography could be used in future studies to see if there is a correlation with cognition or depression. We did not try to see if the mode of treatment for diabetes affected the cognitive functions or depression scores. The other limitations include the absence of controls and the small sample size. The final word on the relationship between diabetes and cognition is yet to be resolved. This negative study points to need for further studies in this area.
